# Decoding Altered Consciousness: An Artery of Percheron Stroke

**DOI:** 10.7759/cureus.55797

**Published:** 2024-03-08

**Authors:** Nadim Jaafar, Rahul Sharma, Neeraj Parkash, Eric P Nolley

**Affiliations:** 1 Internal Medicine, Greater Baltimore Medical Center, Towson, USA; 2 Critical Care Medicine, Greater Baltimore Medical Center, Towson, USA

**Keywords:** ascvd, medical icu, internal medicine, adult neurology, neurology and critical care, posterior circulation stroke, stroke

## Abstract

The artery of Percheron (AOP) is a unique variant of the thalamic and midbrain perforating arteries. It originates from the P1 branch of the posterior cerebral artery (PCA) and supplies the bilateral paramedian thalami (BPT) along with variable contributions to the rostral midbrain. Four infarction patterns have been identified as a result of an AOP stroke, each associated with varying prognostic outcomes. We present an 89-year-old female with an AOP infarction and discuss the associated symptoms, implicated anatomy, and prognosis.

## Introduction

The thalamus, comprised of several unique nuclei, plays a vital role in the central nervous system. It serves as a relay station that transmits sensory, visual, somatosensory, and gustatory signals between various cortical and subcortical regions. Its intricate interconnections implicate it in the regulation of consciousness and alertness, emotions, and memory [1].

Four distinct thalamic structural regions exist based on vascular supply: tuberothalamic, posterior choroidal, inferolateral, and paramedian thalami. In 1973, Gerard Percheron described a unique vascular anatomic variant, the artery of Percheron (AOP). It originates from the P1 branch of the posterior cerebral artery and supplies the bilateral paramedian thalami (BPT), along with variable contributions to the rostral midbrain [2,3]. The prevalence of the AOP remains unknown; however, according to cadaveric studies, it was present in 7% to 11.7% of the general population [4,5].

In this article, we present a rare case of an AOP infarction in an 89-year-old female and discuss the associated symptoms, implicated anatomy, and prognosis. 

## Case presentation

Presentation 

An 89-year-old, previously independent and active female was found at home slouched over on a chair, minimally responsive, and snoring. She was last known well 14 hours prior to hospital arrival. Her medical history was significant for left renal cell carcinoma treated with nephrectomy, breast cancer treated with lumpectomy and hormonal therapy, and hypertension. On arrival to the ED, her temperature was 35.3 °C, heart rate (HR) was 93 BPM, blood pressure (BP) was 180/83 mmHg, respiratory rate (RR) was 28 breaths per minute, and saturation of peripheral oxygen (SpO2) was 96% on two liters of supplemental oxygen. Initial Glasgow Coma Scale (GCS) was 9 (unable to open her eyes to stimulation, brief word responses, and localizing to pain); however, her mentation fluctuated through serial examination from minimally responsive to stimulation to entirely unresponsive. With enough stimulation, she could follow simple commands and answer questions with one or two words. When responsive, she had difficulty opening her eyes and could not direct her gaze toward the examiner. Her pupils were normal in size but sluggish in reaction to light; the cranial nerve examination was otherwise normal. On two brief occasions, she woke up and was oriented to self but not place, time, or situation. Apart from cognitive changes, her neurological exam seemed unremarkable, with strength and sensation preserved in all her extremities. 

Investigations 

The routine complete blood count and comprehensive metabolic panel were normal. The urine toxicology screen and blood-alcohol levels were within normal limits. Further laboratory results are summarized in Table 1. A respiratory pathogen panel was unrevealing. An electrocardiogram revealed a normal sinus rhythm, and an echocardiogram was unremarkable. 

**Table 1:** Laboratory results N: Normal; L: Low; H: High; BUN: Blood urea nitrogen; AST: Aspartate aminotransferase; ALT: Alanine aminotransferase; ALP: Alkaline phosphatase; BAL: Blood alcohol levels; TSH: Thyroid stimulating hormone; HDL-C: High-density lipoprotein cholesterol; LDL-C: Low-density lipoprotein cholesterol

Lab	Result	Interpretation
White blood cells	6.14 10³/µL	N
Hemoglobin	14.5 g/dL	N
Hematocrit	45.3 %	N
Platelets	202 10³/µL	N
Sodium	139 mEq/L	N
Potassium	4.3 mEq/L	N
Chloride	107 mEq/L	N
BUN	20 mg/dL	N
Creatinine	1.06 mg/dL	N
Glucose	92 mg/dL	N
Ionized calcium	4.84 mg/dL	N
AST	18 IU/L	N
ALT	16 IU/L	N
ALP	52.3 IU/L	N
Total bilirubin	0.4 mg/dL	N
Prothrombin time	14.3 Seconds	N
Troponin	< 0.301 ng/mL	N
BAL	< 11 mg/dL	N
TSH	0.27 uIu/mL	L
Thyroxine, free (fT4)	2.02 ng/dL	H
Triiodothyronine, total (T3)	74.59 ng/dL	L
Total cholesterol	220.2 mg/dL	H
Triglycerides	121 mg/dL	N
HDL-C	52.7 mg/dL	N
LDL-C	143 mg/dL	H
Hemoglobin A1C	5.6%	N
Urine toxicology screen	Negative	N

**Figure 1:**
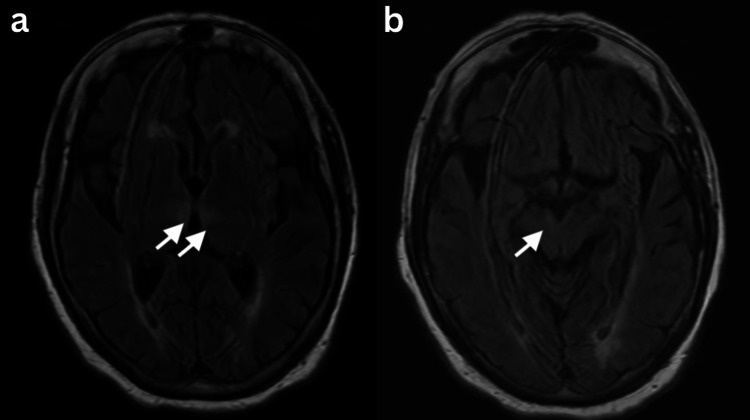
MRI fluid-attenuated inversion recovery (MRI-FLAIR) a) Bilateral thalamic and b) Right-sided rostral midbrain hyperdensity.

**Figure 2:**
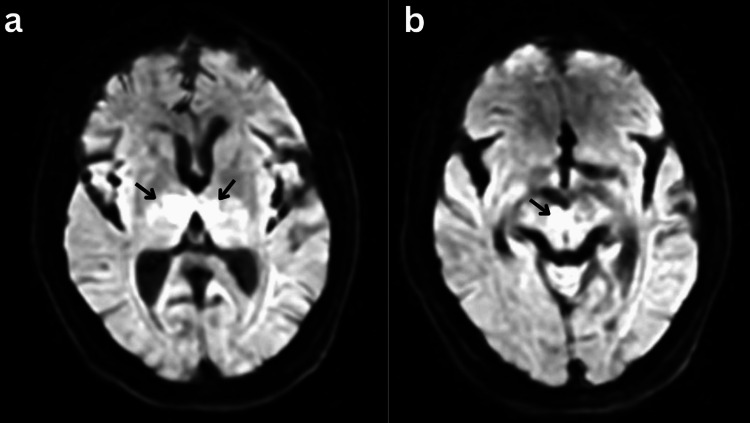
Magnetic resonance diffusion-weighted imaging (DWI) a) Bilateral thalamic and b) Right-sided rostral midbrain restriction.

A CT of the head without contrast was unremarkable. A CT angiography (CTA) of the head and neck with contrast was normal except for a small right A1 artery. An MRI of the brain without contrast revealed a bilateral acute thalamic infarct worse on the right with a slight extension into the right midbrain, suggestive of an occlusion of the anatomic variant AOP (Figures 1, 2).

Hospital course and prognosis 

Given the imaging results and clinical presentation, the patient was diagnosed with a stroke of the AOP involving bilateral thalami and right midbrain. She was not within the thrombolysis window; hence, she was medically managed with aspirin per rectum and blood pressure optimization. Throughout the hospital course, the patient did not demonstrate a meaningful improvement and was discharged to hospice care following the involvement of the palliative care team. Upon discharge, the patient’s clinical condition worsened, and she passed away the following week. 

## Discussion

The artery of Percheron, first identified by Gerard Percheron in 1973, is a rare variant of the thalamic and midbrain perforating arteries. Ischemic strokes in the AOP account for 0.1 to 2% of all strokes and 4 to 18% of thalamic strokes [6]. Non-localizing symptoms and imaging challenges may delay diagnosis, which could ultimately lead to worse outcomes [3]. 

**Figure 3:**
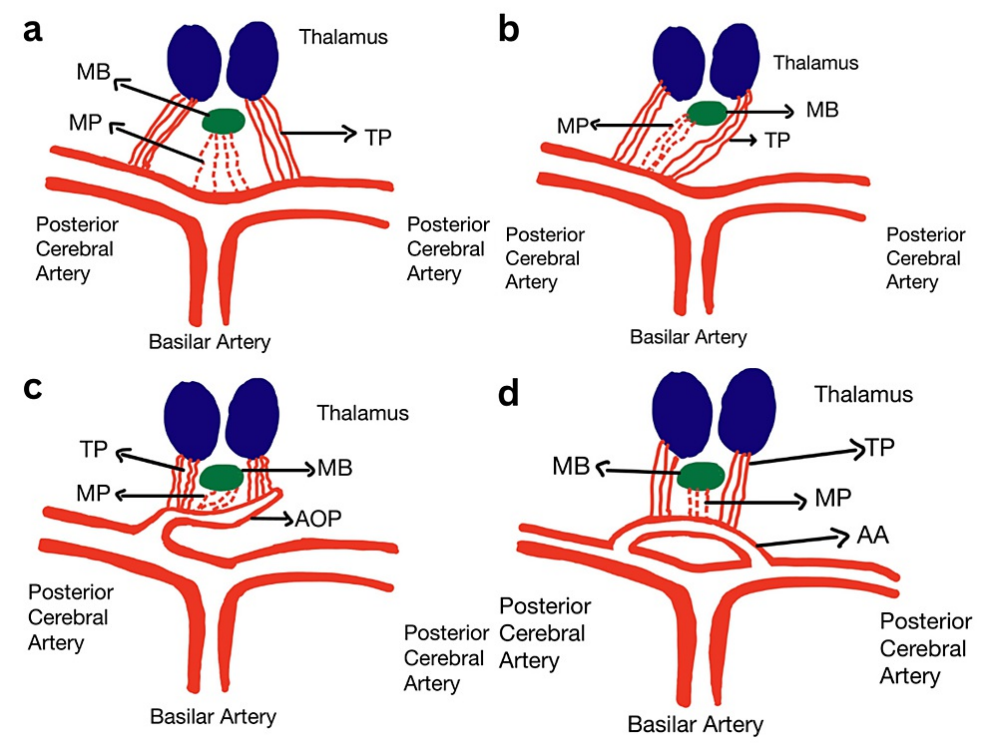
Thalamic and midbrain perforating arteries variations a) Variant I: thalamic and midbrain perforating arteries, each originating separately from the posterior cerebral arteries. b) Variant IIa: thalamic and midbrain perforating arteries originating from one posterior cerebral artery. c) Variant IIb: thalamic and midbrain perforating arteries originating from the artery of Percheron, an arterial trunk arising from the P1 segment of the posterior cerebral artery d) Variant III Thalamic and midbrain perforating arteries arising from the arcade artery, which is an arterial arch that bridges the bilateral P1 segments of the posterior cerebral artery. AA: Arcade artery; AOP: Artery of Percheron; MB: midbrain; MP: Midbrain perforator arteries; TP: Thalamic perforator arteries Image Credit: Author Nadim Jaafar

There are four variants of the origin of the perforating arteries, as shown in Figure 3. In the most common variant, type I, the perforating arteries originate separately from the left and right posterior cerebral artery (PCA). Type IIa entails an asymmetrical origin where the perforating arteries originate directly from one of the PCAs. In Type IIb, perforating arteries arise from the AOP, an arterial trunk originating from P1 of the PCA. Finally, in Type III, the perforating arteries originate from an arterial arc that bridges the P1 segments of both left and right PCAs, known as the arcade artery [6]. 

In a retrospective study by Lazzaro et al., four patterns of AOP infractions were identified. The most common form involves the bilateral paramedian thalamus and rostral midbrain (BPTRM) (43%), followed by the BPT (38%), and bilateral paramedian and anterior thalamic with midbrain (BPATM) (14%). Finally, the least implicated distribution was bilateral paramedian and anterior thalamus (BPAT) (5%) [7]. 

Most patients with an AOP infarction present with progressive drowsiness, ultimately amassing to a coma. However, clinical presentations may vary due to the disparate functions of the implicated structures. Further described manifestations include vertical gaze palsy and ataxia (commonly seen with midbrain involvement), behavior and memory deficits (often associated with disruptions to the hippocampus or its connections), motor dysfunction, and speech difficulties [3,8,9]. It is noteworthy that in clinical practice, associating the symptoms with the underlying infarction/lesion poses a significant challenge to physicians. Our patient presented with alternant drowsiness and coma, difficulty opening eyes and tracking, and memory deficits. Her head CT and CTA were both unrevealing. However, an MRI of the brain revealed bilateral thalamic infarcts, slightly extending to the right-rostral midbrain, consistent with an AOP territory infarct. 

The diagnosis of an AOP infarction follows the stroke algorithms. It has been shown that MRI with diffusion-weighted imaging (DWI) and fluid-attenuated inversion recovery (FLAIR) sequencing offers better chances of early diagnosis and, thus, is regarded as a gold standard investigation [6]. 

According to Zhang et al., patients with BPT and BPAT patterns of AOP infarctions demonstrated a good prognosis and meaningful recovery with appropriate treatment. However, this is not the case with the BPTRM counterpart [8], which was unfortunately present in our patient.

## Conclusions

The artery of Percheron is a rare variant of the arterial supply to the paramedian thalamus and midbrain. A stroke in the AOP territory may go undiagnosed due to non-localizing symptoms and imaging difficulties, which could lead to worse outcomes. Four resultant infarction patterns with varying prognostic outcomes and clinical presentations have been identified. The DWI and FLAIR sequences have been shown to offer better chances of early diagnosis. Further studies are needed to improve AOP detection and awareness. 

## References

[1] Torrico TJ, Munakomi S (2023). Neuroanatomy, thalamus. StatPearls [Internet].

[2] Hiratsuka Y, Kikuchi K, Miki H, Mochizuki T (2012). Evaluation for detectability of the paramedian artery using 3D TOF MRA on 3tesla MRI [Poster]. Poster ECR 2012.

[3] Phate N, Pawar T, Andhale A (2022). Artery of Percheron stroke: a case report with a diagnostic challenge. Cureus.

[4] Kocaeli H, Yilmazlar S, Kuytu T, Korfali E (2013). The artery of Percheron revisited: a cadaveric anatomical study. Acta Neurochir (Wien).

[5] Uz A (2007). Variations in the origin of the thalamoperforating arteries. J Clin Neurosci.

[6] Lamot U, Ribaric I, Popovic KS (2015). Artery of Percheron infarction: review of literature with a case report. Radiol Oncol.

[7] Lazzaro NA, Wright B, Castillo M (2010). Artery of percheron infarction: imaging patterns and clinical spectrum. AJNR Am J Neuroradiol.

[8] Zhang B, Wang X, Gang C, Wang J (2022). Acute percheron infarction: a precision learning. BMC Neurol.

[9] Kichloo A, Jamal SM, Zain EA, Wani F, Vipparala N (2019). Artery of Percheron infarction: a short review. J Investig Med High Impact Case Rep.

